# The intestinal stem cell as a target: A review

**DOI:** 10.1097/MD.0000000000039456

**Published:** 2024-08-23

**Authors:** Nisreen Lutfi Faizo

**Affiliations:** a Department of Clinical Anatomy, Faculty of Medicine, King Abdulaziz University, Jeddah, Saudi Arabia.

**Keywords:** colorectal cancer, intestinal stem cells, Lgr5, Notch, Wnt

## Abstract

Human intestinal epithelium handles several events that may affect health. It is composed of villi and crypts, which contain different types of cells. Each cell type plays an essential role in intestinal functions, including absorption, defense, self-renewal, and regeneration. Intestinal stem cells (ISCs), located at the base of intestinal crypts, play an important role in intestinal homeostasis and renewal. Any disruption in intestinal homeostasis, in which ISCs alter their function, may result in tumor growth. As Wnt and Notch signaling pathways are essential for ISCs homeostasis and for maintaining self-renewal, any defects in these pathways could increase the risk of developing colorectal cancer (CRC). Lgr5^+^ cells have been identified as intestinal stem cells expressing a leucine-rich repeat-containing G-protein-coupled receptor 5 (LGR5), which is involved in the regulation of Wnt signaling. Several studies have reported upregulated expression of LGR5 in CRC. Hence, in this review, we discuss the relationship between LGR5, Wnt signaling, and Notch signaling and the development of CRC, as well as recent therapeutic strategies targeting LGR5, cancer stem cells (CSCs), and the aforementioned signaling pathways.

## 1. Introduction

The human intestinal epithelium consists of a single layer of different cell types arranged in projections (villi) and invaginations (crypts).^[1]^ Each cell type plays a critical role in intestinal epithelial function and homeostasis. Intestinal stem cells (ISCs) are among the most important cell types in the crypt bases.^[2]^ Continuous regeneration of the intestinal epithelium should be carefully balanced between ISCs proliferation and differentiation. ISCs, also called Lgr5^+^ cells, are responsible for homeostatic regeneration of the intestinal epithelium and produce different types of epithelial cells.^[3]^ Transiently amplifying cells progress from the intestinal crypts to the villi, giving rise to secretory lineages or absorptive enterocytes.^[2]^ The colonic epithelium is also driven by the division of Lgr5^+^ stem cells, but it is organized in glands that contain basal crypts with no villus projections.^[4]^

Several signaling pathways are involved in ISCs homeostasis, including the Wnt and Notch signaling pathways.^[4]^ The leucine-rich repeat containing the G-protein-coupled receptor 5 (LGR5) gene has been identified as a specific intestinal stem cell marker and plays a crucial role in Wnt/β-catenin signaling, which eventually catalyzes the self-renewal of ISCs.^[4,5]^ Thus, disruption of Wnt signaling might result in abnormal proliferation of the intestinal epithelium and development of intestinal tumors.^[6]^

Notch signaling is also involved in intestinal epithelial regeneration and ISCs proliferation.^[7]^ Dysregulation of Notch signaling results in loss of ISCs and conversion of amplifying cells into goblet cells.^[8]^ Moreover, defective Notch signaling promotes the growth of colon tumors.^[9]^ We will discuss the Wnt and Notch signaling pathways and Lgr5^+^ cells and their role in the development of intestinal tumors. Targeting Lgr5^+^ cells to treat colon cancer has been a topic of interest, as these cells have demonstrated the potential to be the origin of some intestinal tumors^[10]^; thus, we aim to thoroughly discuss the latest therapeutic strategies that target Lgr5^+^ cells. Different approaches have been used to target Lgr5^+^ cells and have been successful in reducing cell proliferation^[11]^; however, cancer stem cell plasticity remains an obstacle in treating colon cancer.

## 2. Anatomy of intestinal crypts

**Figure 1.**
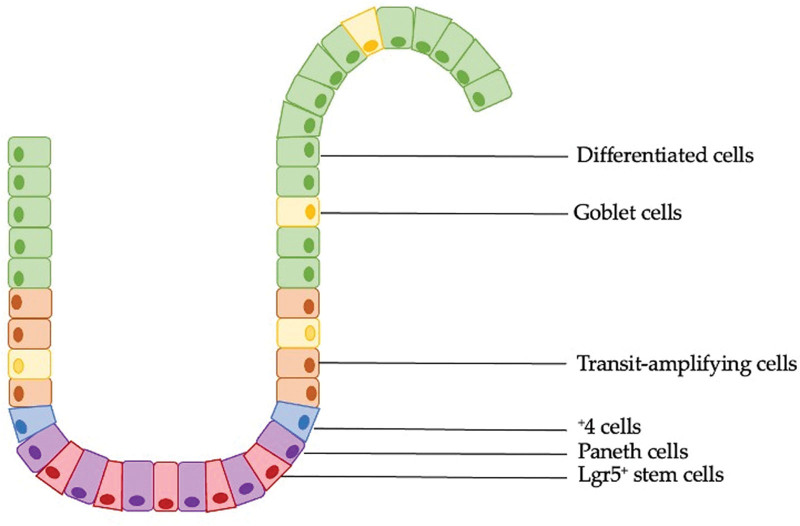
The structure of intestinal crypts. Lgr5^+^ stem cells are located at the crypt base between Paneth cells and are capable of generating transit-amplifying (TA) cells undergo multiple divisions to produce differentiated cell lineages such as columnar cells, goblet cells, enteroendocrine cells and Paneth cells. The ^+^4 cells are able to restore Lgr5^+^ stem cell compartment after an injury. TA = transit-amplifying.

The human intestinal epithelium controls nutrient absorption and provides protection against pathogens. It is arranged in a self-regenerating villus-crypt structure. The intestinal villi, which are finger-like projections into the intestinal lumen, have an increased surface area for nutrient uptake. These villi are composed of differentiated cells, including goblet cells that secrete mucus, enteroendocrine cells that secrete hormones, and absorptive enterocytes.^[12]^ Goblet cells are also present in intestinal crypts. The crypts that are invaginations between villi consist of amplifying cells that are capable of self-renewal of the epithelium.^[13]^ The colonic epithelium is also formed by the constant division of Lgr5^+^ stem cells^[4]^; however, the colonic epithelium has no villus projections, as it is organized in glands that contain basal crypts (Fig. 1). However, several cell types such as Paneth-like cells and enterocyte-like colonocytes, which are present in small intestinal villi, are also present in the upper part of the colonic glands.^[4]^ Periodically activated ISCs are present at the base of the intestinal crypts between fully differentiated Paneth cells, which secrete defensins and lysozyme.^[12,14]^ ISCs replace cells on a daily basis in intestinal crypts.^[15]^ These stem cells enter mitosis, with a cell cycle of approximately 21.5 hours.^[15]^ Crypt-based ISCs are capable of producing progenitors or amplifying cells that undergo multiple divisions to produce differentiated cell lineages as they move up the crypt axis.^[15]^ Cell lineages produced from progeny cells include enterocytes, goblet cells, enteroendocrine cells, Paneth cells, and tuft cells.^[1]^ In intestinal crypts, the stem cell microenvironment (niche) is extremely important for the regulation of progenitor cell functions.^[4]^ The intestinal stem cell niche includes stromal cells, dendritic cells, macrophages, and lymphocytes.^[16]^ The interaction between cells of the niche and ISCs plays a crucial role in the homeostatic development of the intestinal epithelium, the renewal process, and in the protection against overproduction of ISCs.^[17]^ This interaction between niche cells and ISCs can occur through secreted products and/or via direct interaction through the porous basement membrane.^[18]^

## 3. Wnt signaling pathway in ISCs and cancer

**Figure 2.**
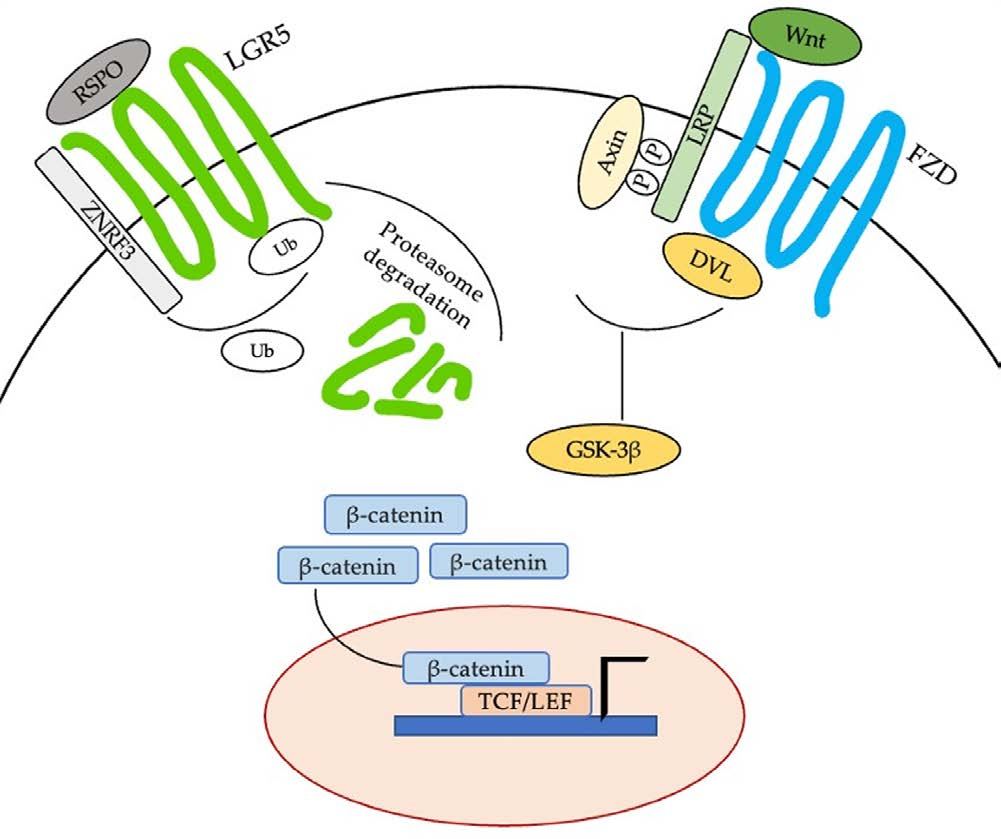
Activation of Wnt signaling through RSPO-LGR binding. When R-spondin (RSPO) binds to leucine-rich repeat containing G-protein–coupled receptor 5 (LGR5) on the cell surface, it recruits the zinc and ring finger 3 (ZNRF3) away from frizzled (FZD) receptors. This results in LGR5 degradation and accumulation of FZD receptors on cell membrane, which increases cellular sensitivity to Wnt ligands and keep Wnt signaling on. When Wnt ligand binds FZD, Axin is sequestered thereby preventing β-catenin destruction which leads to β-catenin accumulation in the cytoplasm. β-catenin is translocated to the nucleus and binds to T-cell factor/lymphoid enhancer-binding factor (TCF/LEF) co-transcription factors that interact with DNA to activate Wnt target gene expression. FZD = frizzled, LEF = lymphoid enhancer-binding factor, LGR = leucine-rich repeat-containing G-protein-coupled receptor 5, RSPO = R-spondin, TCF = T-cell factor, ZNRF3 = zinc and ring finger 3.

The importance of Wnt signaling in the biology of ISCs has been well established. Wnt signaling plays an important role in the physiology of the intestine and is essential for ISCs homeostasis.^[19]^ Wnt proteins are produced by intestinal crypt cells, and the proliferation of transiently amplifying cells depends on the stimulation of Wnt signaling.^[18]^ Although it has been classified as an orphan glycoprotein receptor, LGR5 now is known to have R-spondins (RSPO) as ligands.^[20]^ When RSPO binds to LGR5, it promotes Wnt/β-catenin signaling by working with frizzled (FZD) and LRP5/6 Wnt receptors. This results in the neutralization of the ring finger protein 43 (RNF43) and the zinc and ring finger 3 (ZNRF3) transmembrane E3 ligases that degrade Wnt receptors, leading to a reduction in Wnt signaling and inhibition of Wnt target genes.^[21]^ Thus, the RSPO/LGR5 complex maintains Wnt signaling on which ultimately allows the translocation of cytosolic β-catenin into the nucleus.^[20]^ The nuclear β-catenin, in turn, binds with the co-transcription factors T-cell factor/lymphoid enhancer-binding factor (TCF/LEF) to activate Wnt target gene expression^[22]^ (Fig. 2).

Several studies have been conducted to understand the critical role of Wnt signaling in maintaining ISCs dynamics and intestinal homeostasis.^[23]^ Wnt-depleted organoid cultures could be rescued by mesenchymal cells through providing ISCs with Wnt and RSPO ligands.^[24]^ Also, a study shows that of the ten mammalian FZD receptors, FZD7 deletion leads to disruption of Wnt signaling and loss of ISCs.^[25]^ Deletion of β-catenin showed rapid loss of ISCs and major disruption of intestinal homeostasis.^[26]^ Furthermore, knockdown of TCF4 decreases expression of Wnt target genes which are essential for cell cycle progression.^[27]^ MYC is a Wnt target gene which functions to suppress cell cycle inhibitors; deletion of this gene causes ablation of intestinal crypts.^[27]^ Thus, perturbations to any component of Wnt signaling might result in significant changes in ISCs pool and intestinal homeostasis.

As Wnt signaling pathway regulates several processes including cell proliferation, differentiation and apoptosis, its deregulation has been strongly linked to colorectal cancer (CRC).^[28]^ Aberrant Wnt signaling may promote abnormal ISCs proliferation and initiate tumorigenesis.^[29]^ It has been reported that mutations in RSPO or alteration of its expression may potentiate Wnt signaling, upregulate target genes and induce tumorigenesis.^[30]^ Given the role of LGR5 receptor of RSPO in amplifying Wnt signaling, many studies reported its high expression in CRC.^[20]^ In addition, a recent work has demonstrated that mutation of RNF43 causes proliferation of CRC cells through RSPO.^[31]^ Although activating β-catenin mutations were reported in CRC,^[32]^ they may be considered rare mutations in CRC which could be due to sequestration of β-catenin by E-cadherin to the cell membrane.^[33,34]^ Indeed, more than 90% of CRC cases carry mutations in components of Wnt signaling including Adenomatous Polyposis Coli (APC) and FZD receptor.^[35]^

## 4. Notch signaling pathway in ISCs and cancer

**Figure 3.**
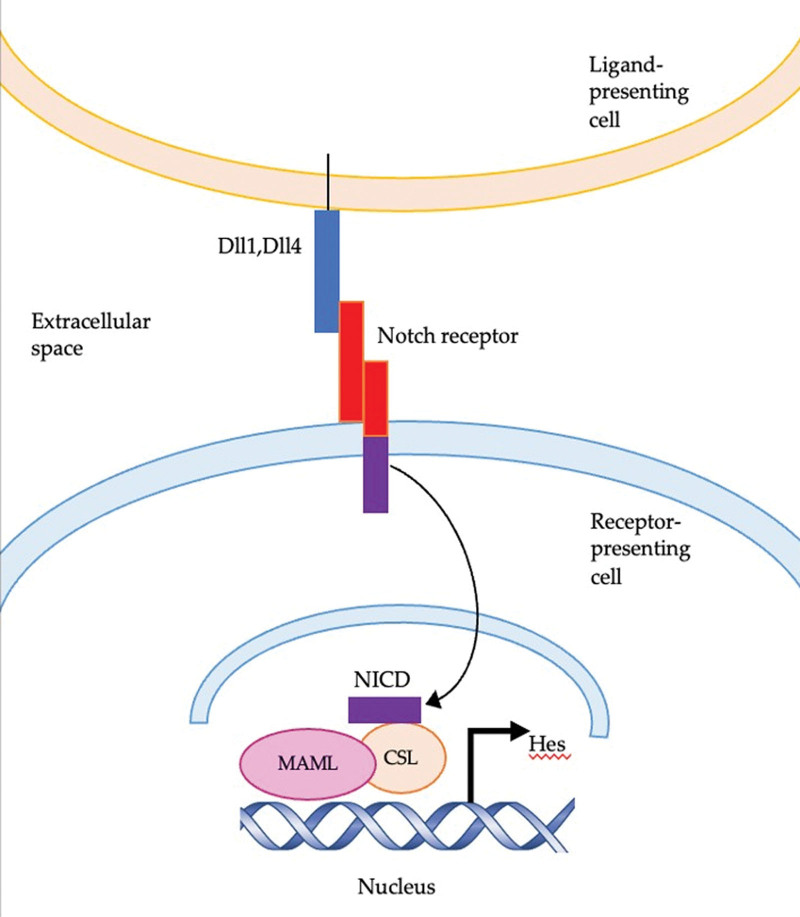
Notch signaling. When the Delta-like Notch ligand (Dll1, Dll4) binds to the Notch receptor at the cell surface, the Notch receptor undergoes a series of proteolytic cleavages which include the removal of the extracellular part of the receptor by ADAM metallopeptidase domain 10 (ADAM10) and the release of a cytoplasmic part of the receptor by gamma-secretase complex. Notch intracellular domain (NICD), which is the active fragment, is subsequently translocated to the nucleus and binds the transcription factor CSL recruiting co-activators such as MAML. This transactivation complex promotes transcription of Notch target genes such as the hairy enhancer of split (HES) family of transcription factors. ADAM10 = ADAM metallopeptidase domain 10, CSL = Chorionic somatomammotropin hormone like 1, HES = hairy enhancer of split, MAML = Mastermind like protein, NICD = notch intracellular domain.

Notch signaling is a conserved pathway that has been implicated in many different processes, including cell differentiation, proliferation, cell fate determination, and death.^[36]^ Notch proteins are transmembrane proteins that function as surface receptors and transcriptional regulators.^[9]^ Notch signaling works by 2 adjacent cells interacting as ligand–receptor binding. This interaction causes a conformational change in Notch receptors, which induces proteolytic cleavage of Notch receptors. The Notch intracellular domain (NICD), which is released into the cytoplasm, enters the nucleus to activate the transcription of target genes. NICD binds to the transcription factor Chorionic somatomammotropin hormone like 1, recruiting co-activators that promote transcriptional activation of target genes^[37]^ (Fig. 3).

Although Notch ligands are mostly expressed in intestinal secretory cells, Notch receptors are highly expressed in intestinal Lgr5^+^ stem cells.^[7]^ As Notch receptors maintain ISCs identity, several studies have reported that dysregulation of Notch signaling causes loss of Lgr5^+^ ISCs, and more secretory cells appear in transient-amplifying cells.^[4]^ It has been demonstrated that when Notch signaling and Wnt signaling are activated, Lgr5^+^ ISCs maintain their proliferative state and self-renewal; however, if Wnt signaling is off, activation of Notch signaling causes suppression of secretory cell fate and leads to differentiation of ISCs into enterocytes.^[7]^ In contrast, when Notch signaling is off, activation of Wnt signaling directs ISCs to differentiate into secretory cell lineages.^[7]^

Several studies have demonstrated the role of Notch signaling in various diseases, including colon cancer. Abnormal Notch signaling promotes colon cancer growth.^[9]^ Moreover, loss of APC, which is a key regulator of Wnt/β-catenin signaling, in addition to activation of Notch signaling in mice, causes the development of intestinal adenomas that could progress to intestinal tumorigenesis.^[38]^ Consistent with this, Notch signaling activity appeared to be increased in APC-deficient tumors, and depletion of the Notch receptor ligand Jag1 led to reduced tumor cell proliferation and reconciled the stem cell niche, which suggests that Jag1 could have an important function in APC-deficient tumors.^[7,39]^ It has been reported that reduced LGR5 expression during intestinal development in mice could have an inhibitory effect on Notch signaling.^[40]^ Moreover, downregulation of Notch signaling in CRC cell lines via knockdown of Notch1 could result in reduction of proliferation, colony formation and tumorigenicity; on the other hand, upregulation of Notch signaling results in increased proliferation and tumorigenicity.^[9,41]^ Therefore, it has been suggested that the decreased proliferation and migration of CRC cell lines due to *LGR5* silencing is related to reduced Notch signaling.^[42]^

## 5. Lgr5^+^ cells

Previous studies identified 2 types of ISCs populations: crypt base columnar cells and ^+^4 cells.^[43]^ Crypt base columnar cells, also called Lgr5^+^ cells, are located at the crypt base between Paneth cells and express the bona fide marker of stemness LGR5.^[5]^ The ^+^4 cells are located at cell position 4 above the differentiated Paneth cells and express other stemness markers such as the polycomb complex protein BMI1.^[1]^ Studies have shown that Lgr5^+^ cells are actively and continuously cycling, whereas ^+^4 cells are slow-cycling or quiescent.^[44]^ Lineage-tracing experiments appear to be less efficient in ^+^4 cells than in Lgr5^+^ cells; thus, Barker et al^[5]^ identified Lgr5^+^ cells as genuine intestinal stem cells. Isolation of Lgr5^+^ cells from the murine small intestine could undergo self-renewal and build self-organizing crypt-villus organoids.^[45]^ LGR5 is a leucine-rich repeat containing G protein-coupled receptor for RSPO ligand that regulates Wnt/β-catenin signaling.^[21]^ It has been reported that RSPO binding to LGR5 in the presence of Wnt leads to an interaction between LGR5 and LRP/FZD receptors of Wnt, which potentiates Wnt/β-catenin signaling.^[46]^ This event occurs through downregulation of the transmembrane E3 ligase enzymes RNF43 and ZNRF3, which release Wnt receptors from the cell surface to regulate Wnt signaling.^[20]^ R-spondins play an essential role in stem cell survival, differentiation and development.^[47]^

Interestingly, NF-κB signaling which is induced by LGR5 plays a role in colon cancer cell survival.^[48]^ LGR5 has been expressed in some human tumors as well as CRC stem cells.^[11,20,49]^ A meta-analysis demonstrated that LGR5 could be a reliable prognostic marker for CRC clinical outcomes.^[50]^ In addition, LGR5 expression in colon cancer tissue organoids is correlated with the tumor stage.^[51]^ Other studies have indicated an association between high LGR5 expression and reduced survival.^[20]^ Although LGR5 expression was upregulated in CRC tissue, there was an insignificant correlation between LGR5 expression and other intestinal stem cell markers, such as ASCL2 and EPHB2.^[52]^ Notably, LGR5 expression is elevated during adenoma-carcinoma transformation and in cancer tissue cells; however, its expression is significantly reduced in metastatic lymph nodes as well as in budding cancer cells, which is not related to Wnt signaling.^[52]^ Moreover, LGR5 overexpression suppresses the proliferation and migration of colon cancer cells in advanced stages of the disease.^[52]^ A study reported that LGR5 could play a role in suppressing tumor growth and migration in the late stages of colon cancer.^[52]^ RSPO1/LGR5 stimulates the transforming growth factor-β (TGFβ) signaling pathway, which inhibits tumor growth and enhances apoptosis in colon cancer cells.^[53]^ Thus, the suppressive role of LGR5 on tumor growth in advanced stages of colon cancer may be attributed to its effect of activating TGFβ signaling.^[53]^

Lgr5^+^ cells express Notch receptors to which Notch ligands of niche cells bind to activate the Notch signaling pathway.^[54]^ Notch signaling plays a crucial role in maintaining ISCs homeostasis, proliferation, differentiation, and determination of cell fate.^[55]^ Pathway analysis demonstrated that the Notch signaling pathway was downregulated during *LGR5* silencing.^[42]^ Ablation of Notch signaling using the γ-secretase inhibitor dibenzazepine causes loss of the entire epithelium, suggesting that Notch could promote the proliferation of absorptive and secretory progenitor cells as well as stem cells.^[7]^

## 6. Targeting Lgr5^+^ cells

LGR5, a biomarker of cancer stem cells (CSCs), appears to contribute to CSCs proliferation by stimulating the Wnt/β-catenin pathway.^[56]^ Increased LGR5 expression has been reported in CRC.^[52]^ It has been demonstrated that Lgr5^+^ cells could be the source of intestinal tumors.^[52]^ In addition, LGR5-induced Wnt signaling plays a considerable role in the development and progression of CRC.^[57]^ Therefore, studies have been conducted to investigate the possibility of therapeutic targeting of LGR5, which may represent a promising strategy to treat CRC.^[20]^ Targeting Lgr5^+^ cells in human colon cancer cells resulted in tumor regression.^[58]^

LGR5 ablation in intestinal crypt stem cells and colon cancer cells causes loss of F-actin, decreases cell–cell adhesion, and disrupts adhesion-associated proteins.^[59]^ A recent study that targeted Lgr5^+^ cells with an antibody conjugated to cytotoxic drugs showed reduced tumor growth and proliferation in colon cancer.^[60]^ Zhang et al^[61]^ reported that monomethyl auristatin E linked with anti-LGR5 antibody-forming antibody-drug conjugate may diminish colorectal cancer tumors. It has also been reported that an RSPO4-furin domain mutant fused to the human peptibody IgG1-Fc and conjugated with cytotoxic drugs could suppress the proliferation of LGR^+^ colon and liver cancer cells in *vitro* and tumor growth in *vivo*.^[62]^ The proprotein convertase furin activates oncogenic precursors involved in the ERK/MAPK signaling pathway, which is activated by KRAS mutations commonly found in CRC.^[63]^ Furin silencing inhibits tumorigenic proliferation of KRAS-mutant CRC cell lines.^[63]^ Additionally, a study revealed that mice-induced colon tumors with KRAS-mutation, in which the Wnt/β-catenin pathway is activated, could be repressed by furin silencing.^[64]^ The latter, in turn, suppresses the tumorigenicity of colon CSCs and causes downregulation of LGR5 and Nanog gene expression.^[64]^ Furthermore, Furin silencing leads to dysregulation of calcium regulators, which may play a role in the tumorigenicity of CSCs, as these regulators are important for stem cell self-renewal and proliferation.^[64,65]^ Thus, furin silencing may have a promising therapeutic effect on Lgr5^+^ cancer cells. Interestingly, a patient-derived xenograft tumor model treated with Rspo1-conjugated doxorubicin liposomes targeting Lgr5^+^ CSCs showed significant tumor tissue necrosis and growth inhibition.^[66]^ This drug delivery system using liposomes conjugated with the LGR5 ligand, which may reach more CSCs and deliver drugs efficiently, could provide an effective new strategy to treat cancer.

Some food components have anti-cancer effects in CRC.^[67]^ Sulforaphane (SFN) is an isothiocyanate obtained from cruciferous vegetables, such as cabbage, cauliflower, and broccoli, and could be a promising anti-cancer agent.^[68]^ Previous studies have suggested that SFN might have a suppressive effect on various CSCs, including pancreatic, breast, and lung CSCs.^[69–71]^ Chen et al^[72]^ showed that SFN could reduce colorectal CSCs properties by suppressing the Tap63α/LGR5/β-catenin pathway. Another study demonstrated that SFN downregulated the expression of some colorectal CSCs markers, including CD44 and LGR5, and inhibited colorectal CSCs via suppression of ∆ Np63α.^[73]^ Curcumin has also been shown to suppress Lgr5^+^ colorectal CSCs by promoting apoptosis and downregulating TFAP2A-mediated extracellular matrix-receptor interaction pathways.^[74]^ Similar inhibitory effects of curcumin have been observed in gastric cancer cells through the promotion of apoptosis and autophagy.^[75]^ Notably, co-treatment with curcumin and polyunsaturated fatty acids not only decreased DNA damage induced by azoxymethane in Lgr5^+^ colon stem cells but also promoted apoptosis of DNA-damaged cells.^[76]^ Furthermore, amorphous curcumin, which has higher solubility and bioavailability, induces apoptosis in CRC organoids and reduces the expression of CSCs markers, such as LGR5 and CD44.^[77]^ Synergistic activity was observed when amorphous curcumin was combined with anti-cancer drugs.^[77]^ Moreover, a study in which curcumin was combined with 5-fluorouracil (5-FU) demonstrated increased apoptosis and reduced proliferation of CRC cells.^[78]^ These studies provide evidence supporting the inhibitory effect of SFN and curcumin on colorectal CSCs, which could be promising therapeutic strategies to treat CRC.

A group of researchers treated 5-FU-resistant colon cancer culture cells with MSI-N1014, a synthesized tetracyclic heterocyclic azathioxanthone, which diminished cell viability and migration and reduced the expression of some oncogenic markers such as mTOR and EGFR, and stem cell markers including LGR5 and β-catenin.^[79]^ This could provide a novel therapeutic strategy to treat 5-FU-resistant colorectal cancer with MSI-N1014, as it demonstrated its therapeutic potential in *vitro* and in *vivo*.^[79]^ To selectively target CSCs, another group of researchers used a fractionated photodynamic therapy technique that significantly suppressed tumor-containing Lgr5^+^ cells with little effect on normal Lgr5^+^ stem cells.^[80]^ The previously mentioned technique involving radiation transfer between cells expressing green fluorescent protein and a rose bengal photosensitizer could demonstrate,^[80]^ in concept, a novel photodynamic therapy to specifically target Lgr5^+^ cells to treat and prevent colon cancer. It is important to note that LGR5 expression is decreased in advanced stages of colon cancer; hence, when RSPO1/LGR5 stimulates TGFβ signaling, it leads to the inhibition of tumor growth and induction of apoptosis.^[53]^

Notch signaling regulates intestinal homeostasis by regulating intestinal stem cells and absorptive cell fate.^[7]^ Although Notch and Wnt signaling pathways have been studied separately, we still do not know how these pathways integrate to regulate ISCs fate and maintain ISCs homeostasis. A critical role of Notch signaling in maintaining ISCs has been demonstrated, which diminishes Wnt signaling output.^[81]^ Targeting Notch signaling and its components in cancer treatment has been established.^[82]^ Inhibition of Notch signaling may be a promising strategy for treating colorectal cancer.^[83]^ Recently, therapeutic antibodies for the treatment of cancer have been widely used.^[82]^ Using selective antibodies that have specific targets provides fewer side effects. For instance, targeting Notch1 or Notch2 receptors using therapeutic antibodies may have anti-angiogenic effect and effectively diminish tumor growth.^[84]^ Furthermore, blockage of Notch receptors with antibodies leads to decreased proliferation of Lgr5^+^ stem cells and reduced LGR5 expression, which is accompanied by upregulation of the Wnt signaling pathway and increased differentiation of secretory cells.^[81]^ In addition, using antibodies to inhibit ADAM metallopeptidase domain 10 (ADAM10), which is overexpressed in colon cancer cells, could reduce Notch signaling and tumor growth; hence, it may provide a promising strategy to treat tumors.^[85]^ The obstacle is that the niche of colon CSCs is similar to that of normal ISCs, which presents challenges for cancer therapeutic strategies, as normal ISCs are essential for maintaining intestinal epithelium homeostasis.

Despite these promising therapeutic strategies, Lgr5^+^ CSCs can convert to Lgr5^-^ cells, which are chemo-resistant and might lead to more aggressive tumors due to CSC plasticity.^[86,87]^ Not only Lgr5^-^ cells comprise the majority of escaping cells from the primary tumor metastasize compared to Lgr5^+^ CSCs, but also acquire LGR5 expression, which appears to be necessary for metastasis outgrowth.^[88]^ However, Lgr5^-^ colon cancer cells can be repressed by MET-targeted antibody-cytotoxic drug conjugates, as these cells depend on the MET/STAT3 pathway to survive.^[89]^ Another study showed that GPR56, a G-protein-coupled receptor involved in cell adhesion, is upregulated in Lgr5^−^ cancer cells which could be a possible mechanism of drug resistance and plasticity in colon cancer cells.^[90]^ The same study reported a significant reduction in tumor growth in CRC cells treated with cytotoxic drugs conjugated to anti-GPR56 monoclonal antibodies.^[90]^ The plasticity of colon cancer cells due to anti-cancer therapy and from tumor growth to metastasis requires the development of therapeutic strategies to cover Lgr5^+^ CSCs and cancer cell plasticity.

## 7. Conclusions

Lgr5^+^ intestinal stem cells located in the intestinal crypt base play an essential role in intestinal homeostasis and renewal. Thus, disruption of intestinal homeostasis and ISCs function could result in intestinal tumor growth. Moreover, defects in Wnt signaling, which is involved in intestinal physiology and ISCs homeostasis, may cause abnormal proliferation and result in the development of CRC. Wnt/β-catenin signaling may be regulated by LGR5, which appears to be highly expressed in CRC. Therefore, several researchers have directed their attention towards targeting Lgr5^+^ ISCs to treat CRC. One of the possible effective strategies is to use antibodies conjugated to cytotoxic drugs, which have been shown to inhibit proliferation and diminish CRC tumors. Moreover, doxorubicin liposomes conjugated with the LGR5 ligand could be an effective drug delivery system to reach more CSCs and treat cancer. Food components, such as SFN and curcumin, alone or in combination with cytotoxic drugs, might have an inhibitory effect on colorectal CSCs, which could also be promising therapeutic strategies to treat CRC. Additionally, abnormal Notch signaling has been linked to colon cancer; hence, targeting the Notch pathway and its components could provide an effective strategy for treating colon cancer. Using therapeutic antibodies against Notch receptors leads to reduced intestinal stem cell proliferation and tumor growth. Despite the previously mentioned strategies, further studies should be performed to selectively target Lgr5^+^ CSCs with little effect on normal cells, in addition to the fractionated photodynamic therapy technique. In addition, the development of new therapeutic strategies or modification of existing strategies to cover Lgr5^+^ CSCs and overcome cancer cell plasticity is required for the effective treatment of CRC at different stages.

## 8. Future Directions

After targeting Lgr5^+^ CSCs, proliferative Lgr5^−^ cancer cells attempt to maintain tumor growth/regrowth to replenish the CSCs pool. Thus, Lgr5^+^ CSCs reappear when cancer treatment is discontinued, resulting in rapid tumor regrowth. Studies should investigate the mechanism underlying CSCs plasticity and tumor regrowth after depletion of Lgr5^+^ CSCs. Since intestinal crypt cells show high plasticity and the ability to re-attain stemness during stress, CRC tissue cells could possibly originate from different types of cell lineages. Although stem cell characterization is supposed to be present in CRC tissue, the heterogenicity of the expression of ISCs marker genes could explain the high plasticity; hence, cancer cells that do not originate from intestinal stem cells may also express ISCs markers to some extent. Therefore, ISCs markers may be expressed in CRC cells without reacquiring full stem cell characterization.

## Acknowledgments

We acknowledge King Abdulaziz University for providing support.

## Author contributions

**Conceptualization:** Nisreen Lutfi Faizo.

**Data curation:** Nisreen Lutfi Faizo.

**Funding acquisition:** Nisreen Lutfi Faizo.

**Project administration:** Nisreen Lutfi Faizo.

**Resources:** Nisreen Lutfi Faizo.

**Software:** Nisreen Lutfi Faizo.

**Supervision:** Nisreen Lutfi Faizo.

**Validation:** Nisreen Lutfi Faizo.

**Visualization:** Nisreen Lutfi Faizo.

**Writing – original draft:** Nisreen Lutfi Faizo.

**Writing – review & editing:** Nisreen Lutfi Faizo.
